# Endovascular Therapy for Isolated Brachiocephalic Pseudoaneurysm

**DOI:** 10.3400/avd.cr.22-00025

**Published:** 2022-06-25

**Authors:** Daisuke Arima, Kazuchika Suzuki, Yumi Kando, Satoshi Akuzawa, Naoyuki Ishigami

**Affiliations:** 1 Department of Cardiovascular Surgery, Fujieda Municipal General Hospital, Fujieda, Shizuoka, Japan

**Keywords:** brachiocephalic artery, pseudoaneurysm, endovascular therapy

## Abstract

In this study, we present a successful endovascular therapy using a small-diameter stent graft for a 73-year-old man who developed asymptomatic pseudoaneurysm of the brachiocephalic artery. An 8F sheath was placed in the brachial artery, and a stiff guidewire was advanced to the descending aorta. The stent graft was delivered to the brachiocephalic artery via the brachial approach. After the initial dilatation, the stent graft was post-dilated to maximum diameter. Final digital subtraction angiography confirmed no endoleak. We believed that endovascular for a brachiocephalic pseudoaneurysm using a small-diameter stent graft might be a minimally invasive and simple method useful in clinical practice.

## Introduction

Cases of isolated brachiocephalic aneurysms are known to be rare; in fact, there is yet no established strategy for their treatment. There have been reports of artificial graft replacement and surgeon-modified stent graft treatments. Artificial graft replacement has been identified as a more invasive technique. However, implanting a surgeon-modified stent graft or iliac extension is also a complicated procedure, as the approach involves the subclavian artery or right common carotid artery while the operation is performed under general anesthesia.

In this study, we present a case of brachiocephalic aneurysm, which we treated with VBX stent graft (W. L. Gore & Associates, Inc., Flagstaff, AZ, USA) via a brachial artery approach under local anesthesia. Here, we describe this successful and minimally invasive procedure. Written informed consent was obtained from the patient for the publication of this report. Because this off-label use of the procedure was quasi-urgent, it was reviewed by an ethics committee after the procedure.

## Case Report

**Figure 1:**
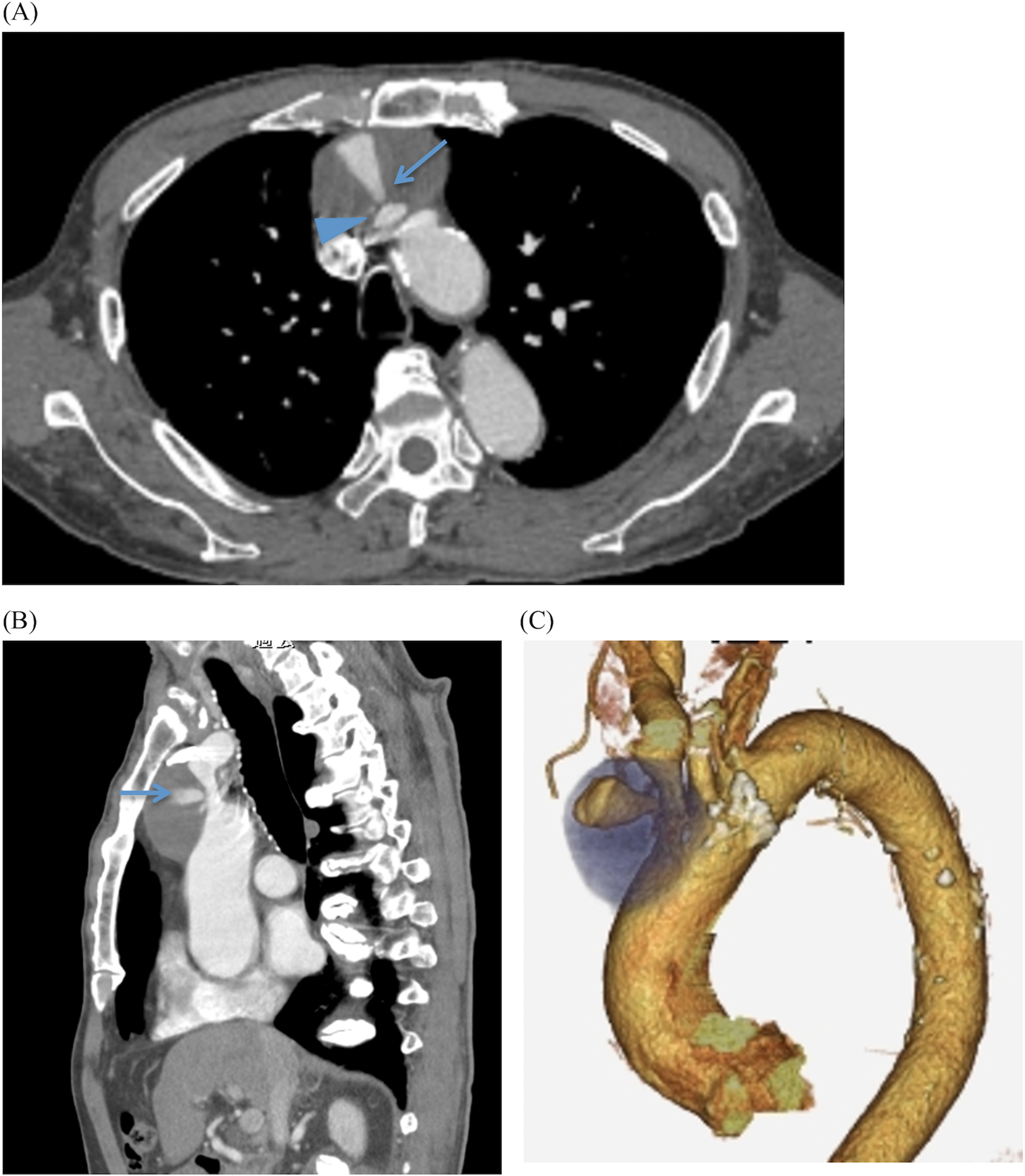
Fig. 1 Preoperative computed tomography images. (**A**) Coronal view showing the compressed brachiocephalic artery (arrowhead) and contrast leakage (arrow) from the brachiocephalic artery to the aneurysm. (**B**) Sagittal view showing the leakage point (arrow) is in the middle of the brachiocephalic artery. (**C**) Three-dimensional constructed image providing an overview of the aneurysm (60×57×38 mm).

A 73-year-old man was referred to our hospital with a chief complaint of diverticular bleeding. The bleeding itself healed, and conservative treatment was planned. A contrast-enhanced computed tomography (CT) scan performed to detect the source of the bleeding has incidentally revealed a brachiocephalic aneurysm. The shape of the aneurysm was noted to be saccular, and much of the interior was thrombosed. There was an area where contrast medium leaked into the aneurysm during the arterial phase, and it was determined to be a pseudoaneurysm (**Figs. 1A** and **1B**). The patient had no chest symptoms and had a previous history of slip and fall trauma of several meters. The patient had a history of hypertension, hyperlipidemia, and cerebral infarction and was being treated medically, including antiplatelet therapy. He presented no fever and no inflammatory signs on blood sampling. The size of the aneurysm was 60×57×38 mm (**Fig. 1C**), and the lumen of the brachiocephalic artery was found to be compressed by the aneurysm. The diameter of the lumen of the brachiocephalic artery was φ 14 mm at the bifurcation of the brachiocephalic artery and φ 9 mm at the narrow part. Morphologically, the risk of rupture was thought to be high; therefore, the patient was treated with semi-urgent endovascular therapy.

### Surgical technique

**Figure 2:**
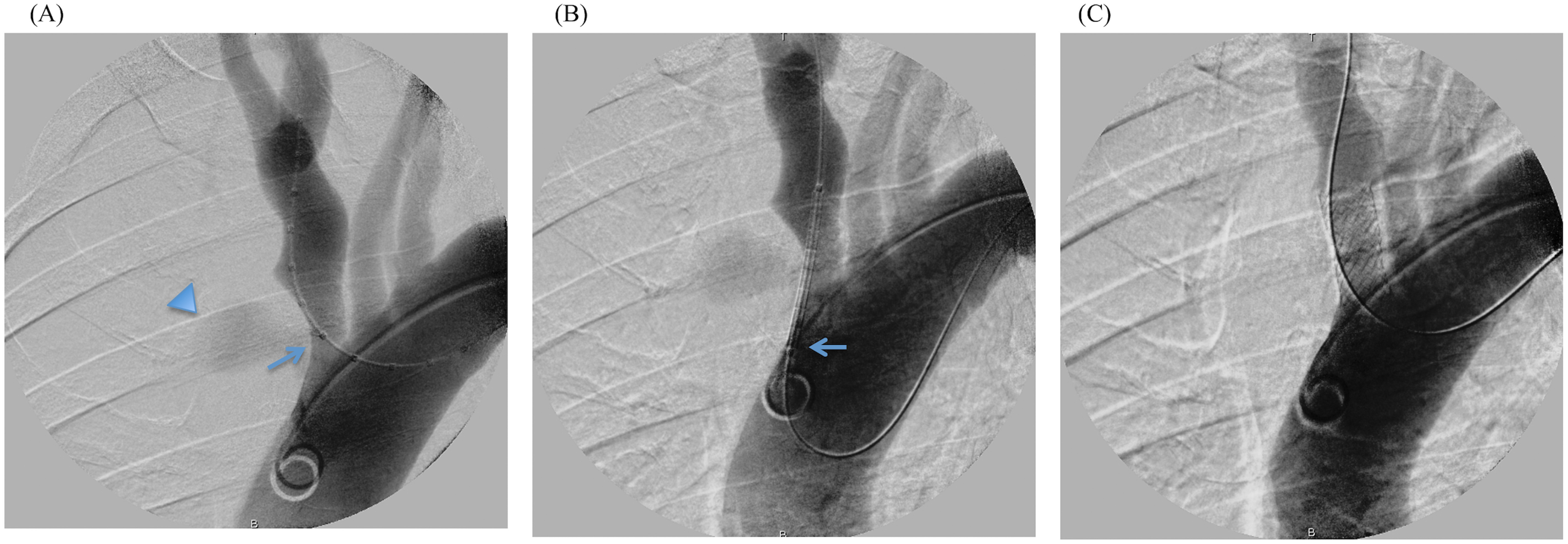
Fig. 2 Angiography on endovascular therapy. (**A**) First digital subtraction angiography shows the contrast leakage (arrowhead) and the leak point (arrow). (**B**) The VBX is delivered to the brachiocephalic artery, and the first proximal stent is placed in the ascending aorta (arrow). (**C**) Final digital subtraction angiography exhibits the proper positioning of the VBX and, moreover, disappearance of contrast leakage.

Under local anesthesia, a 3 cm incision was made in the right upper arm to expose the brachial artery, and an 8F sheath was placed. A 4F sheath was inserted into the right femoral artery. Heparin was thereafter, administered, and the activated clotting time was set at 200 seconds. A 0.035 inch Radifocus Guidewire (Terumo, Tokyo, Japan) and a 4F pigtail catheter were advanced from the right femoral sheath to the ascending aorta. A 0.035 inch Radifocus Guidewire and a 5F pigtail catheter with markers were advanced from the right upper arm sheath to the abdominal aorta. The Radifocus Guidewire was replaced with a 0.035 inch Amplatz Extra-Stiff Guidewire (Cook Medical Inc., Bloomington, IN, USA). The pigtail catheter with markers was returned to the brachiocephalic artery, and treatment length was measured to be 25 mm via digital subtraction angiography (DSA) (**Fig. 2A**). The pigtail catheter with markers was then removed, and the VBX stent graft (BXA113901J) was delivered to the brachiocephalic artery. The implantation position was confirmed by DSA (**Fig. 2B**), and the stent graft was deployed so it would be shortened at maximum dilatation (φ 11× length 34.6 mm by nominal pressure, φ 16×length 25.8 mm by maximum dilatation). Subsequently, a 16 mm Gekira PTA balloon (COSMOTEC, Tokyo, Japan) was used to achieve maximum dilatation. The final DSA showed that the VBX stent graft was appropriately positioned and that the contrast effect in the aneurysm had disappeared (**Fig. 2C**). The catheter was removed, the puncture site of the brachial artery was closed, and the surgery was completed with a closed skin wound. The operation time was 2 h, and total blood loss was 15 ml.

**Figure 3:**
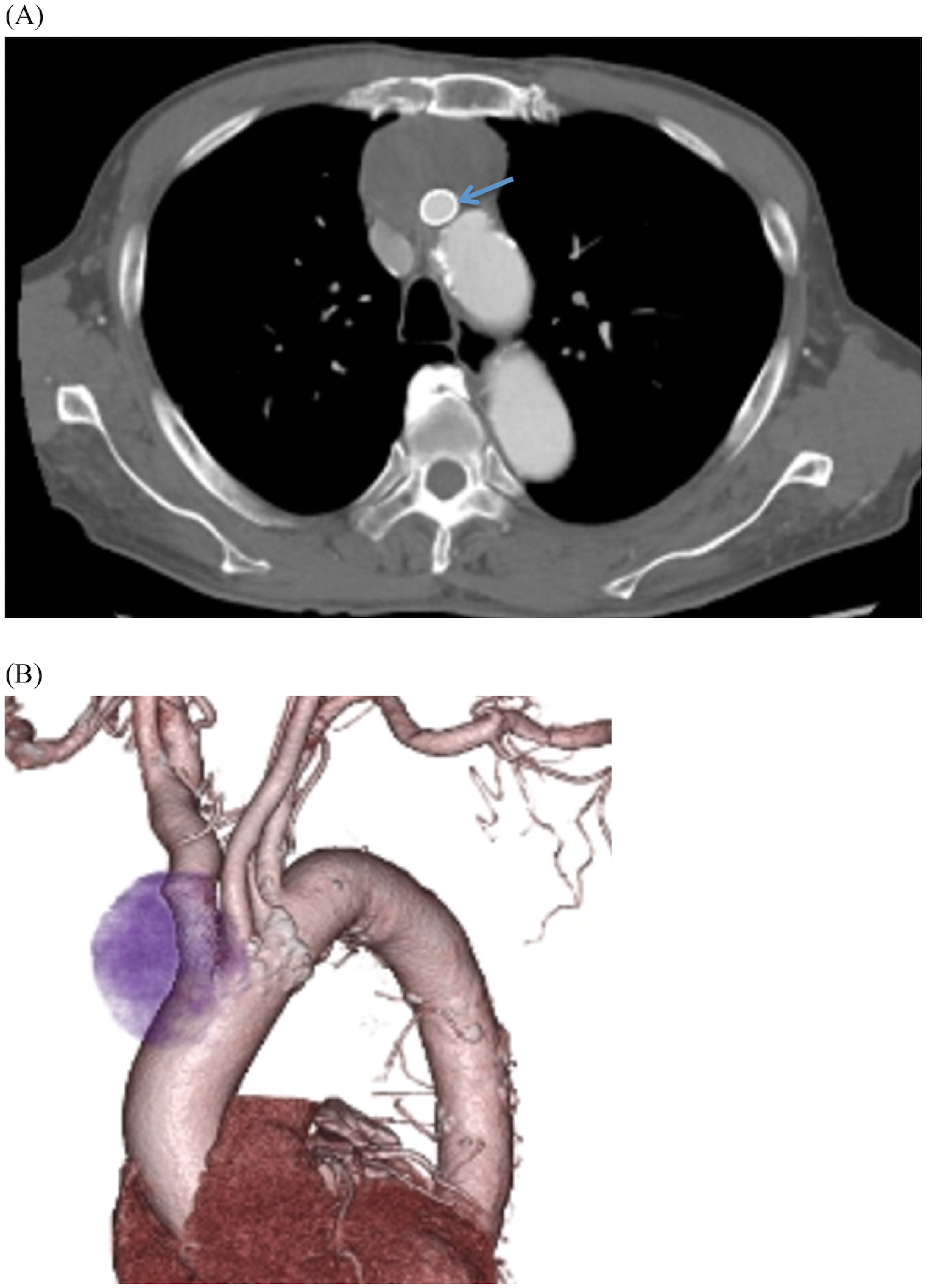
Fig. 3 Postoperative computed tomography images. (**A**) Coronal view showing the disappearance of contrast leakage and uncompressing of the brachiocephalic artery (arrow). (**B**) A three-dimensional constructed image shows the proper position of the VBX.

The patient’s postoperative course was deemed uneventful. A postoperative contrast-enhanced CT scan showed that contrast leakage into the aneurysm had disappeared (**Fig. 3**). The patient is currently undergoing outpatient follow-ups.

## Discussion

Isolated brachiocephalic aneurysms are known to be rare; in fact, most are even asymptomatic. However, depending on the size of the aneurysm, tracheal compression symptoms and rupture can occur.^1)^ Although a few true aneurysms due to atherosclerosis have been reported, cases of pseudoaneurysms due to traumatic, iatrogenic, or infectious events were also found.^2,3)^ However, there remains no standardized treatment for this disease. Case reports have described use of open thoracotomy, endovascular therapy using kissing covered stents technique from the brachiocephalic artery into the common carotid and subclavian arteries, and hybrid treatment using a covered stent and artificial graft.^4–7)^ In this study, we were able to successfully perform endovascular therapy alone in a patient with a pseudoaneurysm of the brachiocephalic artery using the VBX stent to cover the leakage point.

This treatment method has two main advantages. First, this method reduces invasiveness because the surgery is performed under local anesthesia, which is considered to be less invasive than other reported treatment methods. The self-expandable VIABAHN (W. L. Gore & Associates, Inc., Flagstaff, AZ, USA; maximum diameter of 13 mm) is too small for the brachiocephalic artery, while the EXCLUDER iliac extension (W. L. Gore & Associates, Inc., Flagstaff, AZ, USA; minimum dry-seal size of 12F) is too large for the brachial artery. The VBX balloon-expandable stent with a maximum diameter of 16 mm can bridge the gap between the two, and we believe that it is the largest device that can access the brachial artery. One thing to keep in mind is the VBX stent graft has been shortened; therefore, it is technically difficult to deploy it in an implantation position that anticipates this shortening. Shortening occurs evenly from both ends. The VBX should be deployed around the area to be covered and then expanded to the maximum diameter.

Second, leakage is controlled well in the short term. The VBX stent graft has a maximum extension of φ 16 mm, which can accommodate a wider range of arterial diameters that the φ 13 mm of the VIABAHN. Faraj et al. reported a bailout technique using φ 7 mm VBX (from the brachiocephalic artery into the common carotid artery) for iatrogenic brachiocephalic injury.^3)^ Meanwhile, Hu et al. reported using the kissing VIABAHN stent technique for brachiocephalic pseudoaneurysm.^6)^ The short term results of these reports were deemed to be satisfactory. VBX implantation for the brachiocephalic artery was thought to be effective for injury cases and pseudoaneurysms with a clear leak point, as in this case. However, in cases where the neck length is shortened, such as in fusiform aneurysms, treatment may be difficult.

This method has two limitations. First, it cannot be used for brachiocephalic arteries >16 mm in diameter. In addition, the deployment position must have a diameter of at most 11 mm because initial dilatation of VBX stent graft is 11 mm. When the VBX is not applicable, the Endurant Iliac Extension (Medtronic, Minneapolis, MN, USA; minimum outer sheath size, 14F) that has a diameter of 20 mm is trimmed to an appropriate length and thereafter implanted. However, it cannot be approached by puncture through the brachial artery. Second, the number of cases was small, and the long-term outcome of the treatment is yet to be known. Thus, it is necessary to accumulate cases and follow them for an extended period of time. CT follow-up should be performed at 3, 6, and 12 months postoperatively and continued at least annually thereafter.

## Conclusion

Isolated brachiocephalic aneurysm is known to be rare disease, and its standard treatment is yet to be established. Here, we describe the successful use of endovascular therapy in a patient with a pseudoaneurysm of the brachiocephalic artery using a VBX stent graft. With the anatomical conditions satisfied, the method described in our case report might be the most minimally invasive and simple method that could be implemented in clinical practice.

## References

[1] Tahlawi BM, Hassan A, Regal M. Traumatic pseudoaneurysm of the brachiocephalic artery obstructing the airways. Int J Surg Case Rep 2020; 77: 716-8. 33395882 10.1016/j.ijscr.2020.11.064 PMC7718116

[2] Miliauskas S, Benetis R, Zemaitis M, et al. Pseudoaneurysm of brachiocephalic artery mimicking the mediastinal tumor. Respir Med Case Rep 2012; 6: 7-10. 26029593 10.1016/j.rmcr.2012.08.001 PMC3920442

[3] Faraj J, Choudhary A, Ritter JC. Covered stenting as bail-out technique for central venous catheter malposition within the brachiocephalic trunk. Vasc Endovascular Surg 2020; 54: 65-8. 31500541 10.1177/1538574419873175

[4] Mousa AY, Batsides GP, Vogel TR. Delayed presentation of traumatic innominate artery injury. J Vasc Surg 2010; 51: 1014. 19446990 10.1016/j.jvs.2009.03.014

[5] Kota AA, Joseph G, Thomson VS, et al. Endovascular repair of post-traumatic innominate artery pseudoaneurysm. J Vasc Surg 2021; 74: 1015-6. 34425944 10.1016/j.jvs.2020.10.070

[6] Hu SL, Wang CX, Lu HJ, et al. Management of injuries near the innominate artery bifurcation using an accurate kissing Viabahn stent technique. J Int Med Res 2020; 48: 300060520912104. 32393137 10.1177/0300060520912104 PMC7221169

[7] Nagatomo K, Tsutsumi Y, Tsuchiya A, et al. Right proximal common carotid artery injury. J Trauma Acute Care Surg 2021; 91: e18-e20. 34108414 10.1097/TA.0000000000003223

